# Contrasting effects of climate warming on hosts and parasitoids: insights from Rocky Mountain aspen leaf miners and their parasitoids

**DOI:** 10.1098/rspb.2024.2679

**Published:** 2025-03-26

**Authors:** Alisha A. Shah, Emily Hamant, Juan G. Rubalcaba, Beau Larkin, Andrew A. Forbes, H. Arthur Woods

**Affiliations:** ^1^ W.K. Kellogg Biological Station, Department of Integrative Biology, Michigan State University, Hickory Corners, MI 49060, USA; ^2^ Division of Biological Sciences, University of Montana, Missoula, MT, USA; ^3^ Department of Natural Resources and the Environment, Cornell University, Ithaca, NY, USA; ^4^ Faculty of Biological Sciences, Department of Biodiversity, Ecology and Evolution, Complutense University of Madrid, Madrid, Spain; ^5^ MPG Operations, LLC, Missoula, MT, USA; ^6^ Department of Biology, The University of Iowa, Iowa City, IA, USA

**Keywords:** climate change, development, leaf miner, microclimate, upper thermal limit, parasitoid

## Abstract

Because temperature has pervasive effects on biological rates, climate warming may alter the outcomes of interactions between insect hosts and their parasitoids, which, for many host species, constitute the single largest source of mortality. Despite growing interest in parasitoid-host responses to climate change, there are few empirical tests of thermal tolerance differences between non-model lepidopteran hosts and their parasitoids and almost none from mountain ecosystems where warming is occurring more rapidly. We examined the thermal ecology of a host–parasitoid interaction in the Rocky Mountains using wild populations of the aspen leaf miner (*Phyllocnistis populiella*) and a set of previously unknown eulophid parasitoids that attack them. Host and parasitoid development rates were differentially sensitive to temperature. In addition, upper thermal limits of adult parasitoids were lower than those of host caterpillars, and in choice experiments, parasitoids reared at different temperatures showed no plasticity in preferred temperatures. However, when coupled to simulations of leaf microclimates in aspen canopies, these observations suggest, contrary to expectations, that climate warming may potentially benefit parasitoids.

## 1. Background

Temperature plays a crucial role in shaping the outcomes of predator–prey interactions among ectotherms. It affects behavioural factors like speed, strength, agility and reaction times [1–4] which determine how effectively organisms can respond to one another. Temperature also governs the likelihood of interactions by influencing when and where potential interactors co-occur, as well as their capacity for detection and response. For instance, temperature drives the developmental timing of insect hosts and their parasitoids [5], while also modulating the activity patterns [6,7], hunting efficiency [8] and sensory physiology [9] of other types of predators. Prey are similarly affected, with temperature impacting their activity patterns, signal characteristics and ability to detect approaching predators in a timely manner [1]. Together, these temperature-driven effects underpin the dynamics of predator–prey interactions, and may play a critical role determining community response to climate change.

Air temperatures are rising at an unprecedented rate in mountain regions of the world [10], posing a major challenge to montane insect communities that may be more sensitive to warming than lowland communities [11,12]. While some information exists on the thermal physiology of insects inhabiting mountains, such as bees [13] and aquatic insects [14,15], the thermal ecology of parasitoids and their hosts living at higher elevations is mostly unknown. Here, we examined the temperature-dependent dynamics of a parasitoid–host interaction in the Rocky Mountain region, using an eco-physiological approach [16–22]. Parasitoids are an exceptionally diverse group of insects [23] and have shaped the evolution and niches of other insects in almost all terrestrial environments [24]. Their interactions are paramount in structuring insect communities [25], with caterpillars, for example, facing far greater mortality from parasitoids than from all other predators and pathogens combined [26]. Parasitoids therefore play a crucial role as the primary agents of biological control for many Lepidoptera [27–30]. Despite their widespread impact, we have a limited understanding of how non-model parasitoids will respond to warming and whether they differ from their hosts in thermal sensitivity. Generating such information requires experimental data on the thermal ecology of hosts and parasitoids and is a first step in predicting the outcomes of parasitoid–host interactions in the context of climate change [31].

**Figure 1.**
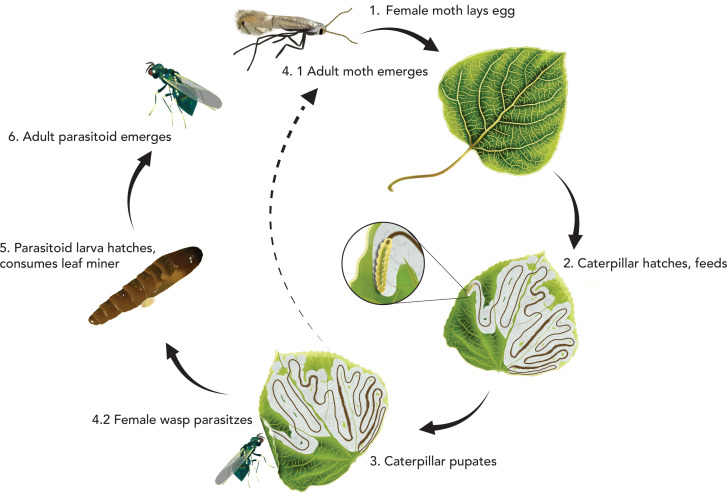
Life cycles of the aspen leaf miner (*Phyllocnistis populiella*) and its parasitoids (Family: Eulophidae), which intersect during specific stages. **1**. In the early spring, overwintered adult female moths lay eggs on newly unfurled aspen leaves. **2**. Caterpillars hatch, feed and grow. **3**. The caterpillar begins to pupate. **4.1** If unparasitized, the pupating caterpillar will eventually emerge as an adult moth. **4.2**. During pupation or shortly before, newly mated adult female parasitoids search leaves for miners on which to deposit their eggs. **5**. Parasitoid larva will hatch, consume the leaf miner pupa and pupate. **6**. An adult parasitoid emerges. It remains unknown if adult parasitoids have additional generations that use other hosts at different times during the year or if they are univoltine, specializing only on the aspen leaf miner. In either case, only one parasitoid generation develops on aspen leaf miner in any single year.

We investigated the effects of temperature on developmental times, thermal preferences, and thermal limits of wild-caught aspen leaf miners (*Phyllocnistis populiella*) and their parasitoids. Aspen leaf miners co-occur with quaking aspen trees (*Populus tremuloides*) in western and northern North America, including in the Rocky Mountains [27,29,30]. Adult moths lay their eggs on leaves (typically a single egg per leaf), and their caterpillars live and feed on the leaf epidermis. During the late larval stages, miners can be parasitized by several species of idiobiont (i.e. host-paralysing) parasitoids in the family Eulophidae (figure 1). Parasitoid eggs develop alongside leaf miner pupae and hatch into larvae, which then consume the leaf miner pupae to complete their development. Although previous research has addressed various aspects of parasitoid foraging [6,32] and the thermal ecology of leaf miners [29,33–38], little is known about the thermal ecology of their interactions.

To assess responses to warming, we focused on two experimentally tractable life stages of the insects: adult parasitoids and larval leaf miners (caterpillars). Interactions between these two life stages—when adult parasitoids seek out mined leaves for caterpillars on which to lay eggs—occur in the spring (i.e. mid- to late June) as air temperatures rise. Under climate change, average spring temperatures are rapidly increasing in this part of the Rockies [39]. The insects are therefore more likely to be faced with potentially dangerously high temperatures in these life stages but may each respond to heat stress differently. We anticipated these differences in thermal limits of parasitoids and leaf miners based on their relative mobility. Even over small spatial extents, differences in microclimatic conditions can be remarkably high [40–42], with strong physiological consequences for ectotherms [33,36,37,40,43–47]. The ability of species to navigate locally available patchworks of microclimates determines their thermal experience and capacity to thermoregulate. For example, a flying insect such as an adult parasitoid, in a given habitat can move from microclimate to microclimate [44], while other mobile animals may limit activity to certain times of the day or night [48] to avoid suboptimal temperatures. The effect of such behavioural compensation is to shield the organism from exposure to the extreme conditions that might select on tolerance [49], and instead maintain body temperatures closer to those at which performance is optimized. A sedentary insect in the same habitat might experience a wider range of temperatures because it is unable to relocate when temperatures become suboptimal. The observation that some organisms can blunt selection on physiological capacities by moving to more suitable locations is commonly described as the ‘Bogert effect’ [50–53].

We predicted that when both immature parasitoid and leaf miner co-occur within a leaf miner pupal chamber, both should exhibit similar tolerance to heat stress because they are similarly immobile. However, when comparing thermal limits of leaf miner larvae to those of adult parasitoids, we predicted that the sedentary leaf miners should tolerate greater heat stress than their parasitoids, which can presumably fly to locations within their preferred thermal window [48,50]. We tested our predictions by measuring development rates of leaf miners and parasitoid pupae exposed to different experimental daytime maximum temperatures, which mimicked cold, normal and future high spring temperatures. We then compared upper thermal limits (LT_50_) in larval miners and adult parasitoids and tested the ability of adult parasitoids to navigate a thermal gradient and locate preferred temperatures (T_PREF_). Because parasitoids were reared at different temperatures, we also tested whether developmental temperature affected adult T_PREF_. Finally, we integrated our experimentally determined leaf miner LT_50_ values, and parasitoid LT_50_ and T_PREF_ values with simulated future microclimatic conditions to predict potential impacts of warming on this host–parasitoid interaction.

## 2. Methods

### (a) Field collection and rearing of parasitoids and miners

We collected 750 aspen leaves with pupal chambers of leaf miners from MPG North, a private research property in Montana, USA (47°31'20" N, 113°40'6" W, elevation 1230 m) in mid-June 2020. To find the leaves, we examined ~100 aspen saplings in a stand covering ~0.5 ha, focusing on mid-sections of these trees (~1.2 m above the ground) and collecting leaves from both peripheral and central parts of the canopy. Leaves were transported to the University of Montana, Missoula, with petioles submerged individually in water-filled picks, which were held upright by poking them into Styrofoam trays. Groups of 150 leaves were then randomly assigned to one of five temperature treatments and placed in temperature-controlled incubators (Percival Scientific I-66LLC8 and I-36LLC8) in which only daytime maxima varied (15, 20, 25, 30 and 35°C) but nighttime temperatures were all 10°C. We used this thermal regime (warm during the day and cool during the night) and a 14:10 L:D cycle to mimic natural temperature and light conditions in the spring at our study site. Once leaves were in incubators, clear plastic drinking cups were secured with tape over each leaf to capture emerging leaf miner moths and parasitoids. Leaves remained in incubators for 50 days and were checked for emerging insects once per day between 9.00 a.m. and 11.00 a.m. Adult insects, i.e. moths (if pupa was non-parasitized) or parasitoids (if pupa was parasitized) were immediately moved into individual, sterile Eppendorf tubes.

### (b) Analysis of developmental rates

All analyses were carried out using R (v. 4.4.0) [54]. To test whether leaf miners and parasitoids developed at different rates across incubation temperatures, we used a linear model that included emergence timing as a response variable, and insect type (leaf miner or parasitoid), rearing temperature and their interaction as predictor variables. Because we had one host species but multiple parasitoid genera and species, we accounted for variation in responses among different parasitoid genera, by running a separate model that included all of the above predictors as fixed effects as well as wasp genus as a random effect. We compared the two models (with and without the random effect ‘wasp genus’) and found that the model without the random effect had a better fit (model with random effect, AIC = 1762.04 versus model without, AIC = 1759.98; dAIC = 2.052). Thus, we opted to use the model that did not control for parasitoid identity.

### (c) Upper lethal temperature (LT_50_)

We measured LT_50_, the temperature at which 50% or more of the individuals died after exposure to acute heat shock, for 47 adult parasitoids. We only used individuals that were reared at 20°C because this treatment most closely approximated early spring temperature at MPG North [37]. Parasitoids were placed in clean, dry, Eppendorf tubes with lids closed tightly, then immersed into a small water bath held at 20°C. Magnets glued to the tube lids attached to a magnetic plate at the bottom of the bath and held the tubes underwater. Air temperature within the tubes was monitored by inserting a type-T thermocouple into a small hole drilled into the cap of an empty tube. Gaps around the insertion point were sealed with epoxy to prevent water from leaking in through the hole, and the thermocouple tip was suspended in the middle of the tube. This tube was immersed into the water bath alongside insect-containing tubes and used as a proxy for temperature readings from all the tubes. We circulated water in the bath to ensure homogenization of water temperature. Because the tubes have thin walls, their internal temperatures equilibrated rapidly with the water bath temperature. Once all tubes containing parasitoids were immersed, the water bath temperature was rapidly ramped (~0.5°C min^−1^) to one of six temperature treatments (*n* ~ 7 individuals per temperature), i.e. 34, 36, 38, 40, 42 and 44°C. We held parasitoids at the heat shock temperatures for 1 h, returned them to room temperature and then assessed mortality after 12 h. We compared heat shock measurements on adult parasitoids to that of larval miners measured in a separate study [37]. Measurement of heat shock in adult parasitoids and larval miners required different protocols because of the different characteristics (larva or adult) and behaviours (sedentary within leaves or active, free-living) of the two types of insects.

Briefly, in our previous study [37], we used custom-built heating devices (SensorSpace, University of Montana Flathead Lake Biological Station, Polson, MT, USA) to measure temperature tolerance of larval leaf miners *in situ* in their leaves because larvae do not survive when removed from the leaf tissue. These devices used Arduino computers to control power to heater tape inside a small, plastic box clamped over a single leaf still attached to the tree. Temperature feedback was provided by a thermistor, and internal temperature gradients were reduced by circulating air using a small computer fan. Heat shock times and temperatures were programmable, and the computer logged realized box temperature once per second. Mine-containing leaves were positioned with larvae directly above the box’s thermistor. Set and measured temperatures usually deviated by <0.5°C. We administered heat shocks between temperatures 38 and 48.4°C for ~1 h. Following the heat shock, each leaf miner’s position in the mine was marked with a Sharpie and photographed (Olympus Tough TG-5). Additional photographs of mines were taken 48 h after the initial photograph and images were analysed to determine whether larvae had moved past the Sharpie dot (indicating survival after heat shock). We first estimated LT_50_ values for leaf miners and parasitoids by fitting a logistic regression where survival was modelled as a function of the heat shock temperature. We used the package *MASS* in R to calculate the median heat shock temperature, i.e. the temperature at which 50% of individuals died. To compare LT_50_ of parasitoids and caterpillars, we fit a logistic regression model to the data of each insect separately, using the *glm* function in R, with survival as the response variable and heat shock temperature as a predictor.

### (d) Thermal preference of adult parasitoids

We examined whether adult parasitoids navigated to preferred temperatures and whether the preference was linked to developmental temperature. First, 1-day-old adult parasitoids were placed in clear plastic flexible aquarium air tubes cut into ~30 cm segments, with ends blocked by plastic stoppers. Tubes were then submerged in water-filled aluminum trenches measuring 35 cm × 0.6 cm × 0.6 cm (L × W × H), which were thermally coupled to an aluminum bar (L 40 cm × W 8.5 cm × H 2.5 cm) with thermal grease. Temperatures at each end of the aluminum bar were fixed by circulating temperature-controlled water through hollowed spaces at each end of the bar, creating a linear thermal gradient along the length of the bar. The cooler end of the bar was 12.5°C and the hot end of the bar was 44.5°C. The temperature within the tubes holding parasitoids, closely matched the temperature of the aluminum bar. The gradient could be activated or deactivated by switching on and off water circulation at each end. We placed a large cardboard box over the experiment and attached a single full-spectrum tube lamp overhead such that light only shone from above and not from the sides of the aluminum bar. This ensured that parasitoid movement wasn’t biased by light from the sides as they tend to orient towards light. We released a single individual into each of the tubes and allowed them 30 min to settle at a location (marked on the aluminum bar in 1 inch We then performed intervals, labelled from 1 to 6) on the gradient that corresponded to a specific temperature. For each individual, we performed a control experiment (i.e. with the thermal gradient deactivated) and a thermal preference experiment (i.e. gradient activated). The order of control and gradient experiments were presented randomly to each insect with ~1 h between experiments.

To determine whether parasitoids showed a thermal preference, we first tested whether parasitoid position was more random (had more spread) in the control experiment than in the gradient experiment using Levene’s test of equality of variances (library *car* in R). We reasoned that if parasitoids made thermal choices, variance in position should be greater in the control experiment, in which there was no choice of temperature, compared to the gradient experiment. Next, for each individual, we calculated the difference in value between positions along the aluminum bar in the control and gradient experiments. We then performed a *t*‐test to determine if the differences in position were significantly different from 0. Finally, we tested whether thermal preference varied as a function of rearing temperature using an analysis of variance (ANOVA). At the end of all experiments, we dried parasitoids at 60°C for 24 h, obtained their dry mass, and wherever possible, identified them to genus using morphology under a microscope. In many cases, parasitoid bodies disintegrated after the drying step and we were unable to identify or sex them. Our analyses therefore do not distinguish thermal responses of female and male parasitoids.

### (e) Microclimate modelling and climate change projections

We investigated the expected impacts of climate change on leaf miners and parasitoids by integrating our LT_50_ and T_PREF_ data with simulated microclimatic conditions under either current or future climate scenarios. To model the microclimatic conditions for leaf miners, we used a biophysical model to compute leaf temperature excess (°C above air temperature) in either sun-exposed or shaded conditions within the canopy (see ‘Leaf temperature model’ in electronic supplementary material, appendix I). The model simulates the heat budget of aspen leaves including absorption of solar radiation, emission of thermal radiation, heat exchanged via convective transfer and heat dissipated due to evaporation of water from the leaf surface. For parasitoids, we used air temperature as the main indicator of the microclimatic conditions experienced by adults. We used the function *micro_global* from the R package *NicheMapR* (v. 3.3.1) [55] to compute air temperature (°C), relative humidity (%), wind velocity (m s^−1^), thermal radiation from the sky (i.e. sky temperature; °C) and direct and diffuse solar radiation (W m^−2^). These parameters were estimated at a height of 1.2 m above the ground in the sun during the months of May, June and July, when parasitoids and leaf miners interact. *NicheMapR* uses long‐term (1960−1991) average monthly values from global climate databases, its associated digital elevation model and physical simulations of solar position to compute hourly estimations of microclimatic variables for the middle day of each month (see details in [56]). We validated the model by comparing predicted and observed distributions of leaf temperature excess (i.e. °C above air temperature [37]) in both sun-exposed and shaded leaves (see ‘Model validation’ in electronic supplementary material, appendix I). We considered the following criteria for validation: (i) the difference between observed and predicted leaf temperature excess (i.e. model’s estimation error) should be smaller than the difference between sun-exposed and shaded leaves; and (ii) the model’s estimation error should be smaller than the leaf miner’s thermal safety margin (i.e. the difference between upper thermal limit and maximum ambient temperature). Predicted temperature excess values met these criteria (see ‘Model validation’ in electronic supplementary material, appendix I; see also §4).

We combined the simulated microclimatic data (hourly estimates of leaf temperature or air temperature for the leaf miner and parasitoid, respectively) with LT_50_ and T_PREF_ using three metrics based on the same units (hours) to facilitate comparison among indicators: (i) thermal risk for leaf miners as the number of hours at which leaf temperature exceeds LT_50_; (ii) thermal risk for parasitoids as the number of hours at which air temperature exceeds the LT_50_; and (iii) thermal window of opportunity for parasitoids as the number of hours at which air temperature is within the range of T_PREF_ ± 1 s.d. We computed these three metrics for sun-exposed leaf temperatures under both current conditions and two future climate change scenarios including the intermediate- (SSP2-4.5) and the high-emissions scenario (SSP5-8.5) for the period 2021−2040 from the Model for Interdisciplinary Research on Climate, MIROC6 (WorldClim 2.0, 52). We computed the metrics of thermal risk and windows of opportunity at the study location in Montana under both current and future climatic conditions.

## 3. Results

### (a) Developmental rates

**Figure 2.**
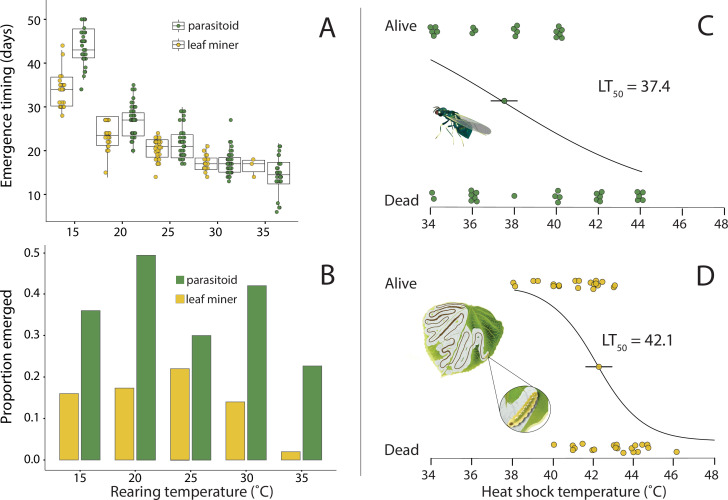
(A) Emergence timing for parasitoids (green) and leaf miners (yellow) emerging from leaves reared at five temperatures. Each circle represents an individual insect. Means and standard error bars are shown. (B) Proportion of parasitoids (green) and leaf miners (yellow) that emerged from leaves at each rearing temperature. (C) LT_50_ for adult parasitoids and (D) larval miners (data obtained with permission from [37]). The parasitoids’ average upper thermal limit was 4.7°C lower than that of larval miners.

From the 750 leaves collected, ~14% (*n* = 107) yielded moths, 36% (*n* = 270) yielded parasitoids and 50% (*n* = 373) yielded no emergences. Both moths and parasitoids developed faster with warming (table 1; figure 2; see electronic supplementary material, figure S3 and appendix II for developmental rate plot), but their developmental rates were differentially sensitive to temperature (significant interaction term in table 1): parasitoids developed slower than moths at the coldest temperature, 15°C, but there was no difference in emergence timing at higher temperatures (25 and 30°C). At the warmest temperature, 35°C, parasitoids emerged faster than moths, though only three moths emerged at 35°C, and they died a few hours later.

**Table 1.** A summary of an ANOVA for the effects of incubation temperature, insect type (parasitoid or leaf miner) and their interaction on emergence timing. Our results indicate significant main effects of temperature and insect type on emergence timing. The significant interaction term indicates that the effect of incubation temperature on emergence timing differs for parasitoids and leaf miners.

parameter	d.f.	sum sq	mean sq	*F-*value	Pr(>*F*)
temperature	1	24 446.8	24 446.8	1152.9	<0.001
insect type	1	932.7	932.7	44	<0.001
temperature: type	1	324.2	324.2	15.3	< 0.001
residuals	367	7781.8	21.2		

Post-experiment inspections of leaves revealed that many moths and parasitoids died as pupae or eclosed as adults but were unable to emerge from the pupal chamber. It is unclear why insects did not emerge from the remaining leaves, but the causes may include disease, nutritional deficiency, drying out of the leaves or stress from field-to-lab transport and handling. Leaf drying could explain the low emergence rate for moths in the 35°C treatment, though the extremely short adult lifespan of the three that did emerge argues more strongly for a developmental or physiological explanation. Among fully emerged parasitoids, genera included *Sympiesis*, *Zagrammosoma*, *Cirrospilus*, *Chrysocharis* and *Clostocerus* (electronic supplementary material, figure S4, appendix II).

### (b) Heat shock (LT_50_)

Caterpillars tolerated higher heat shock temperatures (LT_50_ = 42.1°C) than adult parasitoids (LT_50_ = 37.4°C; figure 2B,C). The logistic regression analysis revealed a significant negative effect of heat shock temperature on survival (*β* = −0.86, s.e. = 0.29, *p* = 0.003 for leaf miners and *β* = −0.25, s.e. = 0.11, *p* = 0.02 for parasitoids) indicating that higher temperatures generally decreased the likelihood of survival for both insects. Upper lethal temperatures were higher than the upper developmental temperatures for both insects, but especially caterpillars (42.1°C versus ~35°C). This result is expected, as insects typically survive higher temperatures when they are exposed for shorter periods of time [57].

### (c) Thermal preference in adult parasitoids

**Figure 3.**
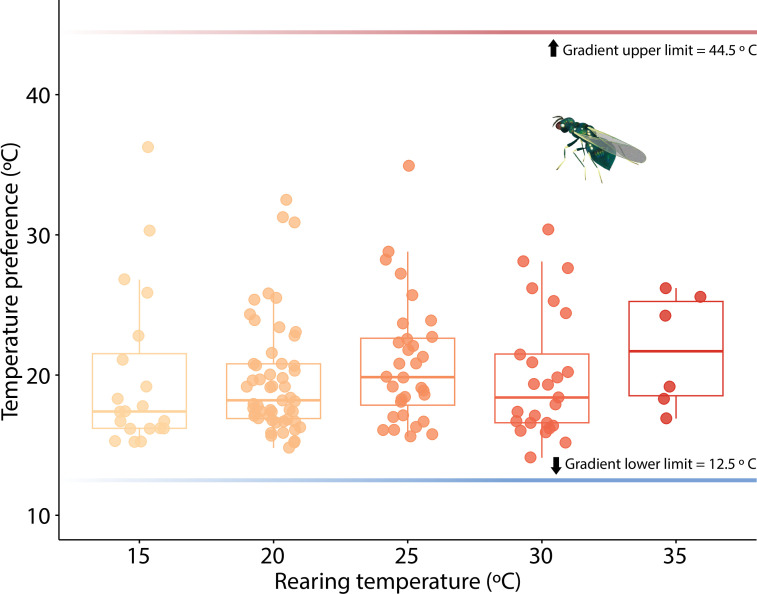
Thermal preference of adult parasitoids. The *y*-axis indicates single temperature measurements taken at the location of each parasitoid (represented by each dot) after 30 min of exposure to the thermal gradient. The temperatures along the gradient ranged from 12.5°C to 44.5°C. Parasitoids preferred cooler temperatures along the gradient (mean = 20.1°C, s.d. = 4.5) and this preference did not vary significantly among parasitoids that experienced different rearing temperatures.

Compared to parasitoids in the control treatment (no thermal gradient), those exposed to a thermal gradient had significantly lower variance in position (Levene’s test for paired samples: *F*_(1, 270)_ = 14.51, *p* < 0.005; electronic supplementary material, figure S5, appendix III). Differences in position between gradient and control experiments were significantly different from zero, indicating that when exposed to the thermal gradient, parasitoids chose higher position values (closer to the cold side of the aluminum bar; *t*_(134)_ = 10.036, *p* < 0.001). The average T_PREF_ of parasitoids exposed to the gradient was 20.1°C, 95% CI (19.3, 20.9; figure 3). T_PREF_ did not vary with rearing temperature (*F*_(1,134)_ = 0.72, *p* = 0.40).

### (d) Microclimate modelling and climate projections

**Figure 4.**
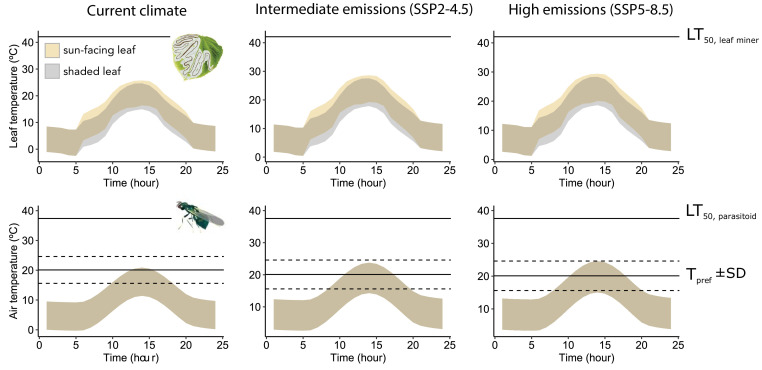
Predicted leaf and air temperatures between May and July in the study area under either current or future climatic conditions. Top panel: leaf temperatures will not exceed the LT_50_ of aspen leaf miners, in sun-facing (peach shading) or shaded leaves (grey shading). Bottom panel: air temperatures will not exceed the LT_50_ of parasitoids; however, warming will increase the amount of time parasitoids experience their preferred temperatures (T_PREF_).

Maximum leaf and air temperatures in sun-exposed conditions were 25.7°C and 20.9°C, i.e. below LT_50_ for the leaf miner (42.1°C) and its parasitoids (37.4°C). Therefore, thermal risk (hours exceeding LT_50_) was zero (figure 4). The parasitoids’ window of opportunity (hours when air temp was within T_PREF_ ± 1 s.d.) was 5.3 h daily. Warming will not raise thermal risk for the leaf miner or parasitoids under intermediate- or high-emissions scenarios (maximum leaf temp 28.6°C and air temp 23.8°C in intermediate-; 29.4°C and 24.6°C, respectively, in the high-emissions scenario). However, the parasitoids’ window of opportunity is predicted to increase to 6.7 h daily in intermediate-emissions and to 8 h in high-emissions scenario (figure 4).

## 4. Discussion

The fate of species under climate change—whether they persist, thrive, relocate or face extinction—depends on both their physiological responses to abiotic stressors and the effects of these stressors on the outcome of interspecific interactions [12,58,59]. In our study, we investigated the thermal sensitivity of wild-caught aspen leaf miners and their previously undocumented parasitoids, which interact in aspen trees across high-elevation and high-latitude regions of North America. These regions, which are experiencing a rapidly warming climate, remain largely understudied in terms of lepidopteran hosts and their parasitoids. Our findings demonstrate that leaf miners and their parasitoids exhibit different thermal sensitivities across the life stages in which they interact. These differences appear to be linked to stage-specific movement and thermoregulatory capabilities and therefore may have important implications for the interaction under a changing climate.

Higher temperatures stimulated more rapid development in both leaf miners and parasitoids but with relatively greater effects on parasitoids. Although leaf miners developed and emerged more rapidly than parasitoids at low temperatures, their emergence became more synchronized with parasitoids at higher temperatures. In addition, leaf miners experienced higher mortality rates at daytime temperatures above 30°C, though the few that survived developed rapidly and emerged as adults. While the ecological consequences of these developmental responses to warming remain unclear in this system—partly because we lack knowledge about whether the parasitoids utilize multiple hosts across generations within a season or are univoltine—such variation in host and parasitoid responses could broadly disrupt phenological synchrony, with potential consequences for community composition [12,60–62]. For example, the loss of these interactions may release herbivores from parasitoid control, leading to increased herbivory [11]. In this system, however, we found that several parasitoid species target the same host. If these species are differentially sensitive to warming during other life stages not tested here, warming may alter the timing of their life cycles and synchrony among species.

We also observed differences between larval hosts and adult parasitoids in their ability to survive short-term exposure to high temperatures (LT_50_). Parasitoid LT_50_ was 4.7°C lower than that of leaf miners. Similar findings for other groups of parasitoids and hosts suggest this pattern of varying tolerance to heat stress may be widespread [5,19,63–65] and differences in thermal traits may reflect evolution in response to patterns of high-temperature exposure in nature. Leaf miners likely evolved higher temperature tolerances due to the strong chance of hatching on sun-exposed leaves, which experience significantly higher temperatures (on average, 5.12°C above air temperature) than shaded leaves (1.35°C above air temperature; electronic supplementary material, table S2) [37]. In contrast, adult parasitoids move through microclimates generated by tree canopy vegetation to locate food, mates and mined leaves for oviposition [6]. Given their small size (<2 mm long), their body temperatures equilibrate rapidly with the surrounding conditions [66]. Thus, although parasitoids may frequently face stressfully high temperatures during hot, sunny days in the spring, their ability to fly away and exploit shaded areas may shield them from selection for higher heat tolerance, which is consistent with the Bogert effect [49–51,67,68].

Given our results on LT_50_ of parasitoids and caterpillars, it is tempting to conclude that parasitoids are more vulnerable to warming [5,65]. However, such a conclusion does not account for the thermal experiences of parasitoids and hosts within their microclimates. When we incorporated both sunlit and shaded conditions within ecologically realistic temperature ranges for the two insects, the conclusion was altered: parasitoids and leaf miners may be *equally* susceptible to warming.

This alteration likely reflects that leaf miners experience relatively warmer microhabitats compared to parasitoids during key parts of the host–parasitoid interaction. Because sun-facing leaf temperatures can become much warmer than air, leaf miners have an estimated thermal safety margin (i.e. the difference between the upper thermal limit and the maximum ambient temperature [69]) of 16.4°C (42.1–25.7°C), during the day’s warmest time. Parasitoids, however, have lower LT_50_ but also experience relatively cooler temperatures. Therefore, they have a similar thermal safety margin of 16.5°C (37.4–20.9°C). The estimated thermal safety margin of the leaf miner was much higher than the deviation between predicted and observed leaf temperature excess in sun-exposed leaves (predicted were ~1.6°C above observed values; see ‘Model validation’ in electronic supplementary material, appendix I) thus supporting the accuracy of the model. Still, this 1.6°C deviation indicates that the thermal safety margin may in fact be slightly underestimated. Although both leaf miners and parasitoids are unlikely to encounter lethally high temperatures in northern regions of their ranges, climate warming will decrease the thermal safety margin of both species and could render them more susceptible to extreme events [34]. Furthermore, our work did not account for sub-lethal effects of warming, which may have different consequences for parasitoids and leaf miners long before upper lethal limits are reached, potentially uncoupling interactions [64,70]. Indeed, in another study of thermal vulnerability in (aquatic) insects, important transitions across stages in the life cycle failed at temperatures well below critical thermal limits [71]. Still, our findings underscore the importance of integrating physiological data on thermal tolerances with known or estimated patterns of thermal exposure in the field [44,72].

Adult parasitoids consistently preferred ~20°C irrespective of taxonomic identity or highest temperature experienced during development. In fact, T_PREF_ was slightly *higher* than the average ambient springtime temperatures in this region of the Rockies, indicating that parasitoids may benefit from initial warming in this region. Currently, parasitoids encounter several hours daily around T_PREF_. Our study shows that future warming will extend the duration of this preferred thermal window. This could have a positive outcome in the short term, as many insects in high latitude and high elevation regions are often constrained by cold temperatures [73–75], but see [76]. However, while increased mean temperatures in our study area will provide adult parasitoids with longer periods of preferred temperatures, it is uncertain whether this will be advantageous during extreme weather events. For instance, their responses to heatwaves and heat domes, which are increasing in frequency and intensity [77], are still unknown. Other parasitoids, such as *Cotesia congregata*, exhibit higher mortality rates than their caterpillar hosts (*Manduca* spp.) under simulated heat waves [70,78]. Whether such findings are similar in non-model species such as those in our study system requires further investigation. Additionally, the relationship between T_PREF_ and the temperature at which performance is optimized (T_OPT_) is not well understood. Untangling this relationship is challenging and needs not only further experimental work, but a better understanding of *which* performance traits to target in experiments. This challenge is prevalent in most research linking the physiological ecology of wild populations with their responses to climate change.

To generalize these findings, we call for further work in several areas. First, our understanding of the life cycle and natural history of these parasitoids remains incomplete. Given the short growing season in these mountain ecosystems, it is plausible that most or all of the parasitoids are univoltine; however, they may also have multiple short generations per year. Furthermore, their overwintering strategies are unclear. Like other temperate parasitoids [79], they likely overwinter as adults, emerging in synchrony with the hatching of their leaf miner hosts. However, warming winters and earlier springs could disrupt this synchrony [80], leading to phenological mismatches that reduce parasitization rates. Thus, phenological mismatches may arise from warmer winters that alter diapause termination or from warming springs and summers that affect the period after parasitization. These multifaceted and complex effects of seasonal warming warrant further investigation. Second, although our experiments and measurements were resource-intensive, we still were unable to account for the thermal experiences, and potential differential thermal sensitivities, of other life stages. Without a more complete understanding of the responses of other life-stages to warming, we are unable to predict with more certainty how the outcome of such interactions will be altered under climate change. Thus, future efforts should focus on understanding variation in thermal sensitivity across the egg, pupal and adult stages of both insects. Third, our experiments imposed relatively short-term changes to thermal environments; additional work on other species pairs should explore the effects of longer-term exposure to altered temperatures [81]. Fourth, we could not link parasitoid performance (i.e. rates of parasitization) to thermal conditions in the field due to logistical challenges at the start of the COVID-19 pandemic. However, these links would provide key information for how the outcome of the interaction is affected by warming. Finally, it is important to account for effects of heat stress on the host plant (i.e. quaking aspen), which provides essential nutrients that shape caterpillar quality. Indeed, climatic effects on plants may have an indirect effect on rates of parasitism and parasitoid success [82] and must be incorporated in future research. Together, these efforts will allow us to more accurately predict the future of these interactions in a changing world [83] and the consequences for the host plants on which the interactions occur.

## Data Availability

All experimental and modelling data as well as R code are freely accessible via Dryad [84]. Supplementary material is available online [85].
